# A case of severe basilar invagination and AAD, corrected using the technique of DCER—distraction, compression, extension, reduction (with spacer + universal reducer)

**DOI:** 10.3171/2020.4.FocusVid.20138

**Published:** 2020-07-01

**Authors:** P. Sarat Chandra, Mohit Agarwal

**Affiliations:** Department of Neurosurgery, All India Institute of Medical Sciences (AIIMS), New Delhi, India

**Keywords:** basilar invagination, atlantoaxial dislocation, distraction, compression, C1–2 spacers, craniovertebral junction

## Abstract

The author has described his own technique of DCER (distraction, compression, extension, and reduction) to reduce and realign the deformity and relieve spinal compression (indicated in congenital anomalies with occipitalized C1 arch). In addition, he developed special C1–2 spacers and a universal reducer. Here, a 30-year-old male with severe BI (20 mm, above the clivus) with AAD underwent the technique of spacer placement (distraction) followed by cable reduction (leading to compression and extension at the occiput–C1–C2 region). Another short example is presented where an 8-year-old boy (severe BI, AAD with posterior fossa dermoid) underwent additional correction—C2 forward translation and excision of the dermoid.

The video can be found here: https://youtu.be/XIMpkYjxgRk

## Transcript

**0:20** We present a case of severe basilar invagination and atlantoaxial dislocation which was treated by relieving the compression, correcting the deformity, and realigning using the technique of distraction, compression, extension, and reduction, abbreviated as DCER. This is a unique technique developed by us using a posterior-only approach. During the presentation, we will also be showing some unique implants and reduction devices used to perform this surgery (Chandra et al., 2013, 2014, 2017, 2019; Chandra, 2015a; Chandra, 2015b; Chandra and Goyal, 2015; Joaquim et al., 2018).

These are my disclosures.

**0:55** Clinical details of the patient. A 30-year-old male presenting with progressive spastic quadriparesis. Examination revealed power of 3–4/5 with a graded sensory loss below the C3 dermatome.

**1:08** These are x-rays, with active flexion and extension views which show occipitalized C1 arch, atlantoaxial dislocation, and possibly basilar invagination, the latter of course not clearly made out on the x-rays. One should also notice the tendency for the cervical spine to become hyperlordotic in such cases.

**1:27** CT scan of the patient with sagittal reconstruction. This shows severe basilar invagination in the midsagittal plane. The parasagittal section shows the severe angulation of the true joint. In order to compensate this, one may notice the formation of a pseudojoint posteriorly as labeled here which forms between the occipital-cervical footplate and proximal superior surface of the C2 pars. Pseudojoint is a concept first described by us and we will describe it a little bit more shortly.

**1:58** This is the CT scan with 3D reconstruction. We do it to look for the position of the vertebral artery. As it can be seen here, it is not present over the joints.

**2:10** MRI shows severe compression of the cord at the cervicomedullary junction.

Here, I would like to briefly explain the concept of the technique DCER. DCER stands for distraction, compression, extension, and reduction. It is based on the principle of first distracting the joints using a spacer, as shown in the lower left image. This corrects the basilar invagination but not the AAD or cervical deformity. Following this, compressive forces are applied between the O–C1 and C2, as shown by red arrows, and this leads to correction of both residual basilar invagination and atlantoaxial dislocation. Thus, the fundamental principle is to use the spacers first as a distractor and then as a fulcrum is very similar to the concept of Archimedes proposing to lift the Earth using it as a lever mechanism (Chandra et al., 2013, 2014, 2017, 2019; Chandra, 2015a; Chandra, 2015b; Chandra and Goyal, 2015; Joaquim et al., 2018).

**3:03** The chief indication of DCER is in congenital etiologies where there is occipitalization of C1 arch and is especially associated with severe atlantoaxial dislocation (AAD) and basilar invagination (Chandra et al., 2017, 2019; Joaquim et al., 2018; Chandra, 2015).

**3:19** Since we are using the spacer as a pivot, and using the lever mechanism to reduce AAD and basilar invagination, we prefer an occipital purchase and not C1. We have found a three-point occipital–C2/3 or C3/4 fixation very good.

**3:37** Enclosed are some of our seminal publications, which we encourage the readers to go through to have a better idea about the concept and techniques (Chandra et al., 2013, 2014, 2017, 2019; Chandra, 2015a; Chandra, 2015b; Chandra and Goyal, 2015; Joaquim et al., 2018).

**3:57** DCER, as has been shown in our studies, not only relieves the cord compression but also corrects the deformity. It has shown to correct the alignment of the entire spinal column.

**4:00** Now, here I would like to briefly explain the concept of pseudojoint. We have shown in our studies that as the severity of basilar invagination and AAD increases, the true joint becomes progressively vertical. The weight of the cranium now thus gets supported by the formation of a pseudojoint, which is an articulation between the inferior surface of O–C1 footplate and proximal superior surface of the C2 pars. The lower image shows the finding which have seen very consistently that whenever the true joint becomes vertical, the existence of the pseudojoint is an anatomical surety (Chandra et al., 2014, 2017, 2019; Joaquim et al., 2018; Chandra, 2015; Chandra, 2015).

**4:42** This slide shows the surgery performed. Foramen magnum decompression, placement of 16- to 18-mm-sized spacers, technique of DCER, and occipitocervical fixation.

**4:56** The patient is positioned prone. I prefer a horseshoe. I prefer to attach a Gardner Well’s traction just a weight of about 3 kg. An exposure is made from occiput till C4.

As can be seen here, the left fused C1 arch is being drilled, the left C2 ganglion is being exposed. The left C2 ganglion is being retracted superiorly to expose the joint.

**5:32** The foramen magnum is now being drilled. This is important to avoid any cord compression during the reduction maneuver. As can be seen here, the foramen magnum was very deep and causing severe compression. In addition, I also prefer to release the bands.

**5:47** The right joint is now being exposed. I prefer to use a variable impedance bipolars, in this case being from Sutter company, and this provides excellent hemostasis.

The next step is to disarticulate the joints. This is performed using a periosteal elevator by first inserting it and then turning it by 90°. It is important to disarticulate and loosen the joints bilaterally.

**6:16** We are now inserting the specially designed spacers, first on the left side and then on the right side. Since the basilar invagination is very severe here, we use progressively increase the size of spacers till we reach to the final size.

**6:37** It is to be understood that the joints that we are freeing are also partly the pseudojoints; hence, we also call this technique as extra-articular distraction. We request the viewers to read our papers to further understand this.

**6:52** These spacers are biconvex in shape, hence can slide into joints easily and are available from 4-mm height till 22 mm. They have a hollow cavity into which bone grafts or hydroxyapatite can be filled.

**7:07** We are now placing the C2 pars and C3 lateral mass screws. The video is shown in 2× speed.

**7:20** We are now showing the reducing system. This consists of a temporary occipital plate, which has two components, a wire reducing system and a C2 translator which pushes the C2 forward. The wire is specially made of braided stainless steel wire attached to a special tensioner. In this case, we have used only the cable wire compression system.

**7:52** So here, we first fix the temporary occipital plate. Here we fix two plates, one small one anteriorly and another posteriorly. This allows the compression and extension of O–C1 and C2 take place only in a vertical direction.

**8:10** Now we are looping the special braided stainless steel wire around the C2 spine and then passing it through the hole of occipital plate. This is now passed through a crimp and then attached to a specially designed wire tensioner.

**8:26** As we are using the wire tensioner to pull the wire, the wire is now tightened between the C2 spine and the occipital plate.

**8:35** One can now notice the superior movement of C2 spine. Thus, the initial movement is that of compression that firmly impacts the spacers. This is followed by extension between the O–C1 and C2 joint leading to reduction of the AAD and also basilar invagination.

**9:01** This is the intraoperative x-ray. The O-arm shows that enough reduction was not achieved, which was expected.

**9:15** So, now we remove the earlier spacers and place larger-sized spacers, in this case was 18 and 16 mm. Increasing the height of spacers also provides a better advantage for the lever mechanism and thus is also more effective to reduce AAD.

I prefer to use the periosteal elevator for a “railroading” mechanism, which allows me to insert the spacers easily. This is now inserting the spacer on the right side.

**10:01** Now, after larger-sized spacers are inserted bilaterally, we perform the technique of compression and extension at the O–C1 and C2 joint using the braided stainless steel cable again.

**10:17** Now we have achieved complete reduction as compared to the preop imaging.

**10:28** Now we are contouring the rod to achieve the standard three-point occipitocervical fixation. Adequate bone graft mixed with hydroxyapatite should be placed to achieve good bone fusion.

**10:46** Postoperative CT shows excellent reduction of both AAD and basilar invagination. Also note the optimal correction of the cervical hyperlordosis, which will also correct the complete spine alignment.

**10:54** We will now show a short video on the use of C2 translator. This is an 8-year-old child presenting with severe spastic quadriparesis.

**11:26** CT scan showed a severe basilar invagination. The true joint, as can be seen here, is completely vertical and the whole weight of the cranium is supported only by the pseudojoint. There is also a backward listhesis of C2 over O–C1 complex. Patient also had a rare association of a posterior fossa dermoid.

So, we placed the spacers within the pseudojoint, a technique described by us called extra-articular distraction and then we performed the DCER. In addition, we used the C2 translator to push the C2 forward to correct the listhesis (Chandra et al., 2017, 2019).

**11:40** Here, I would like to briefly describe what happens in cases where the true joint becomes vertical and the whole weight is transmitted by the pseudojoint. Now, in mild to moderate AAD with basilar invaginations where the true joint is not completely vertical, just a correction using compression and extension technique with only a cable will suffice, as shown in the first row. So, following compression and extension, the center of gravity, abbreviated here as COG, align completely with each other after compression and extension. However, when the true joint becomes vertical, and the basilar invagination is very severe, as in this case, there is also a backward listhesis of C2 over O–C1, as shown in second row. Thus, just compression and extension movements do not align the center of gravities of O–C1 and C2 complexes. Here, we would require an additional pushing force over C2 to move it anteriorly, as shown in the last row to align the center of gravities (Chandra et al., 2017, 2019).

**12:47** Thus, in the reducing system that we devised, we use an additional C2 translator as shown by the red down-pointing arrow. Thus, by using the C2 translator we are adding a T to the technique of DCER; thus, now it becomes DCETR, which stands for distraction, compression, extension, translation, and reduction.

**13:14** So, in this short video, the spacers have already been placed; the lateral mass screws are already in situ. The occipital plate is in position as in previous case. We pass the wire as in previous case, and in this case we have placed a C2 clamp, which I prefer to place in pediatric cases.

Now, I am placing the C2 translator, which has a fulcrum over the occipital plate. A screw is passed now through the hole in the structure of the occipital plate. Tightening this screw now thus pushes the C2 anteriorly.

**13:49** This shows the mechanism of compression and extension being performed by the tensioner and the C2 translation which is being performed by the screwdriver. Both the motions should be performed simultaneously, and this allows a complete reduction.

**14:09** In this case, we have used a specially designed spacer screw which attaches the spacer to the occipitocervical rod, thus providing a rigid fixation.

**14:21** Postoperative CT scan shows complete reduction, and the dermoid was also removed in the same sitting using small occipital craniectomy and endoscopic assistance.

Thank you very much for watching this.
